# Changes in quality of life 1 year after intensive care: a multicenter prospective cohort of ICU survivors

**DOI:** 10.1186/s13054-024-05036-5

**Published:** 2024-07-25

**Authors:** Lucy L. Porter, Koen S. Simons, Stijn Corsten, Brigitte Westerhof, Thijs C. D. Rettig, Esther Ewalds, Inge Janssen, Crétien Jacobs, Susanne van Santen, Arjen J. C. Slooter, Margaretha C. E. van der Woude, Johannes G. van der Hoeven, Marieke Zegers, Mark van den Boogaard

**Affiliations:** 1 Department of Intensive Care, Radboud University Medical Center, Geert Grooteplein Zuid 10, 6525 GA Nijmegen, The Netherlands; 2 Department of Intensive Care, Jeroen Bosch Hospital, ’s Hertogenbosch, The Netherlands; 3 Department of Intensive Care, Canisius Wilhelmina Hospital, Nijmegen, The Netherlands; 4 Department of Intensive Care, Rijnstate Hospital, Arnhem, The Netherlands; 5 Department of Anesthesiology, Intensive Care and Pain Medicine, Amphia Hospital, Breda, The Netherlands; 6 Department of Intensive Care, Bernhoven Hospital, Uden, The Netherlands; 7 Department of Intensive Care, Maas Hospital Pantein, Boxmeer, The Netherlands; 8 Department of Intensive Care, Elkerliek Hospital, Helmond, The Netherlands; 9 Department of Intensive Care, Maastricht University Medical Center, Maastricht, The Netherlands; 10 Departments of Psychiatry and Intensive Care Medicine, and UMC Utrecht Brain Center, University Medical Center Utrecht, Utrecht University, Utrecht, The Netherlands; 11 Department of Neurology, UZ Brussel and Vrije Universiteit Brussel, Brussels, Belgium; 12 Zuyderland Medical Center, Department of Intensive Care, Heerlen, The Netherlands; 13 Department of Intensive Care, Amsterdam University Medical Center, Location AC, Amsterdam, The Netherlands

**Keywords:** Quality of life, Critical care outcomes, Prognosis, Patient-reported outcome measures

## Abstract

**Background:**

With survival rates of critical illness increasing, quality of life measures are becoming an important outcome of ICU treatment. Therefore, to study the impact of critical illness on quality of life, we explored quality of life before and 1 year after ICU admission in different subgroups of ICU survivors.

**Methods:**

Data from an ongoing prospective multicenter cohort study, the MONITOR-IC, were used. Patients admitted to the ICU in one of eleven participating hospitals between July 2016 and June 2021 were included. Outcome was defined as change in quality of life, measured using the EuroQol five-dimensional (EQ-5D-5L) questionnaire, and calculated by subtracting the EQ-5D-5L score 1 day before hospital admission from the EQ-5D-5L score 1 year post-ICU. Based on the minimal clinically important difference, a change in quality of life was defined as a change in EQ-5D-5L score of ≥ 0.08. Subgroups of patients were based on admission diagnosis.

**Results:**

A total of 3913 (50.6%) included patients completed both baseline and follow-up questionnaires. 1 year post-ICU, patients admitted after a cerebrovascular accident, intracerebral hemorrhage, or (neuro)trauma, on average experienced a significant decrease in quality of life. Conversely, 11 other subgroups of ICU survivors reported improvements in quality of life. The largest average increase in quality of life was seen in patients admitted due to respiratory disease (mean 0.17, SD 0.38), whereas the largest average decrease was observed in trauma patients (mean -0.13, SD 0.28). However, in each of the studied 22 subgroups there were survivors who reported a significant increase in QoL and survivors who reported a significant decrease in QoL.

**Conclusions:**

This large prospective multicenter cohort study demonstrated the diversity in long-term quality of life between, and even within, subgroups of ICU survivors. These findings emphasize the need for personalized information and post-ICU care.

*Trial registration:* The MONITOR-IC study was registered at ClinicalTrials.gov: NCT03246334 on August 2nd 2017.

**Supplementary Information:**

The online version contains supplementary material available at 10.1186/s13054-024-05036-5.

## Background

ICU survivors’ quality of life (QoL) is generally lower than that of the general population [1]. Additionally, with survival rates of critical illness increasing, patient reported outcomes, such as QoL, are becoming an important factor in decisions regarding ICU treatment [2–4]. However, as most studies do not take pre-ICU QoL into account, it is unknown to what extent this reduced QoL is attributable to critical illness [5].

To study the long-term impact of critical illness and ICU treatment on patient outcomes, the MONITOR-IC study was initiated in the Netherlands. This multicenter prospective cohort study provides both a baseline measurement and follow-up data up to 5 years after ICU admission. This study has shown that physical, mental, and cognitive symptoms after ICU discharge can negatively affect patients’ QoL [6].

However, due to the heterogeneity of the ICU population, general data alone may not be sufficient. By gaining insight into the long-term outcomes of subgroups of ICU survivors, we can better inform patients with different characteristics about their expected long-term outcomes after an ICU admission. This may also facilitate the incorporation of long-term outcomes, such as QoL, in ICU treatment.

Therefore, the aim of this study was to explore QoL before and 1 year after ICU admission in different subgroups of ICU survivors.

## Methods

### Study design

The MONITOR-IC study, an ongoing multicenter prospective cohort study (ClinicalTrials.gov: NCT03246334, registered on August 2nd 2017), was approved on August 23rd 2016 by the research ethics committee of the Radboud university medical center, the Netherlands (2016–2724) and conducted in accordance with the declaration of Helsinki. Each participant, or their legal representative, provided written informed consent [7] . This study was reported in line with the Strengthening the Reporting of Observational Studies in Epidemiology (STROBE) guideline for cohort studies (E-Appendix 1) [8].

### Study population

Patients were included if they were 16 years of age or older and admitted for ≥ 12 h to the ICU of one of the eleven participating hospitals between July 2016 and June 2021. Patients were excluded if they had a short life expectancy (≤ 48 h), or did not speak the Dutch language.

### Data collection

Patients were asked to complete self-administered paper-based or online questionnaires regarding their health status before hospital admission. If patients were unable to fill in the questionnaires themselves, proxies were asked to perform this task. When possible, elective surgical patients received the questionnaires preoperatively. Other patients, or their proxies, received the questionnaires during admission and were asked to fill in the questionnaires as soon as possible, recalling their QoL on the day before hospital admission.

1 year after ICU admission, patients received a paper-based or online follow-up questionnaire regarding, among others, QoL. In case of non-response, study participants received two reminders.

Data on admission type, admission diagnosis, and co-morbidities were retrieved from the Dutch National Intensive Care Evaluation registry [9, 10].

### Outcome

Outcome was defined as change in QoL, measured using the EuroQol five-dimensional (EQ-5D-5L) questionnaire, and calculated by subtracting the pre-admission EQ-5D-5L index score from the EQ-5D-5L index score 1 year after ICU admission [11]. The EQ-5D-5L questionnaire is a validated questionnaire and is commonly used for measuring health-related QoL after critical illness [12–14]. Each of its five questions represents a dimension of health-related QoL: mobility, self-care, usual activities, pain/discomfort, and anxiety/depression. Each dimension has five levels, ranging from no problems (1) to severe problems (5). The Dutch EQ-5D-5L index ranges from -0.446 to 1, with a higher score indicating a better health-related QoL [15]. The reference value of the Dutch general population aged ≥ 40 years is 0.85 [15].

Considering that previous studies have identified a minimal clinically important difference (MCID) of 0.08, an improvement in QoL was defined as an increase in EQ-5D-5L index score of ≥ 0.08, a deterioration in QoL was defined as a decrease of ≥ 0.08, and an unchanged QoL was defined as a delta EQ-5D-5L index score < 0.08 [16, 17].

### Subgroups

Patients were grouped by their primary admission diagnosis, using the Acute Physiology and Chronic Health Evaluation (APACHE) IV diagnosis system, resulting in 6 main groups and 22 subgroups, based on previous studies and expert opinion [1, 18–22]. The list of diagnoses can be found in Table E1.

### Statistical analysis

Only ICU survivors who completed both baseline and 1-year EQ-5D-5L questionnaires were included in the analyses. Descriptive statistics were performed to assess differences between responders and non-responders, using the chi-square test, independent-sample t-test, or Wilcoxon rank sum test, whenever appropriate. Continuous data were presented as means with standard deviation (SD) or medians with first and third quartile expressed as interquartile range (IQR), depending on their distribution. Categorical data were presented as numbers and percentages.

Since not all data were normally distributed, statistical differences between EQ-5D-5L index scores before hospital admission and 1 year after ICU admission were assessed using the Wilcoxon signed ranks test.

A *p*-value of < 0.05 was considered statistically significant for all analyses. Analyses were performed with R software, version 3.6.2 (R Foundation for Statistical Computing).

## Results

**Fig. 1:**
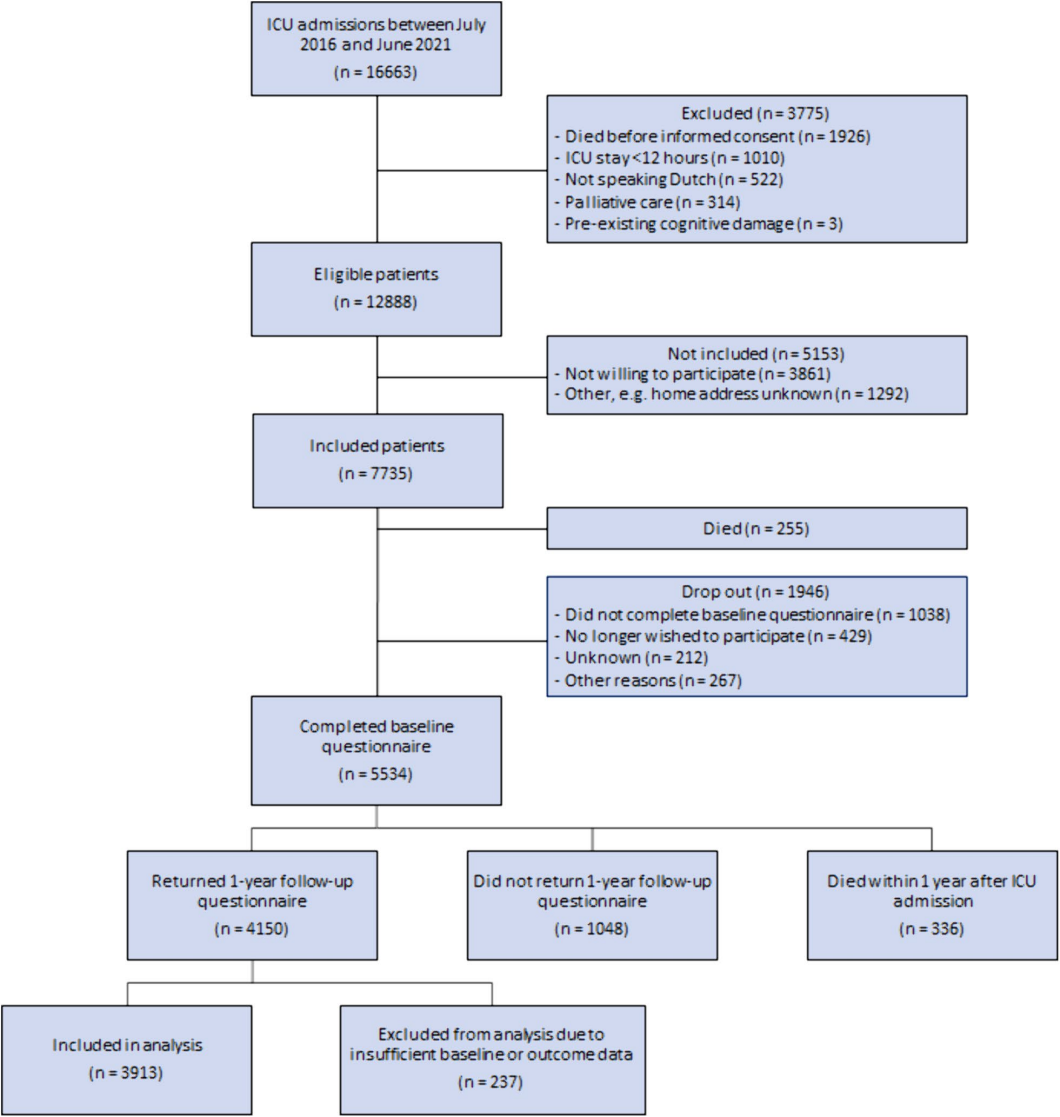
Flowchart inclusion procedure

Of 12888 eligible patients, 7735 (60.0%) patients were included, of whom 5534 (71.5%) responded to the baseline questionnaire. 5198 (93.9%) patients survived until 1 year after ICU, of whom 3913 (75.3%) completed both baseline and follow-up questionnaires, and were therefore included in analysis (Fig. 1).

The non-responders to the 1-year follow-up questionnaire (n = 1048) differed significantly from the responders. Among others, non-responders were younger, had more comorbidities and reported a lower pre-admission QoL (Table E2).

The age of included patients varied between groups with the youngest group being patients admitted after trauma (mean 53.9, SD 18.3) and the oldest group consisting of patients admitted after cardiovascular surgery (mean 66.2, SD 9.3) (Table 1). This variability was also seen in the number of patients with comorbidities, with the prevalence of chronic obstructive pulmonary disease ranging from 2.7% in patients admitted due to trauma, to 17.8% in patients admitted due to respiratory disease. Patients admitted due to cardiovascular surgery had a short ICU length of stay (median 1.0 day, IQR 0.8–1.7) and hospital length of stay (median 8.4 days, IQR 6.4–11.6). In contrast, patients admitted due to a respiratory disease had a median ICU length of stay of 7.6 days (IQR 3.0–16.3) and median hospital length of stay of 16.5 days (IQR 9.0–28.5).

**Table 1:** Patient characteristics

Variable	Cardiovascular (n = 587)	Cardiovascular surgery (n = 1464)	Respiratory (n = 675)	Neurological (n = 186)	Trauma (n = 184)	Other (n = 817)
Baseline questionnaire completed by proxy, *n (%)*	67 (12.3)	69 (5.1)	46 (11.9)	63 (34.2)	55 (30.9)	80 (10.5)
Sex: Female, *n (%)*	197 (33.5)	319 (21.8)	217 (32.2)	92 (49.5)	54 (29.4)	339 (41.5)
Age (yrs), *mean (SD)*	62.6 (13.0)	66.2 (9.3)	61.3 (11.2)	57.3 (14.9)	53.9 (18.3)	61.6 (13.9)
Comorbidities, *n (%)* Chronic obstructive pulmonary disease Diabetes Chronic renal insufficiency Immunological insufficiency Cardiovascular insufficiency Metastasized neoplasm	44 (7.5) 67 (11.4) 26 (4.4) 60 (10.2) 18 (3.1) 12 (2.0)	103 (7.0) 214 (14.6) 27 (1.8) 42 (2.9) 58 (4.0) 2 (0.1)	120 (17.8) 97 (14.4) 22 (3.3) 92 (13.6) 7 (1.0) 7 (1.0)	8 (4.3) 21 (11.3) 2 (1.1) 19 (10.2) 3 (1.6) 2 (1.1)	5 (2.7) 14 (7.6) 1 (0.5) 6 (3.3) 0 (0.0) 1 (0.5)	36 (4.4) 76 (9.3) 36 (4.4) 111 (13.6) 12 (1.5) 103 (12.6)
Mechanical ventilation within 24 h of ICU admission, *n (%)*	313 (53.3)	1405 (96.0)	473 (70.0)	90 (48.4)	118 (64.1)	382 (46.8)
Vasoactive medication in first 24 h of ICU admission, *n (%)*	334 (56.9)	1165 (79.6)	354 (52.4)	72 (38.7)	72 (39.1)	394 (48.2)
APACHE IV score, *mean (SD)*	67.9 (29.8)	53.2 (15.1)	61.3 (18.2)	47.5 (21.8)	50.6 (20.7)	49.4 (18.5)
ICU length of stay (days), *median (IQR)*	2.1 (1.1–4.6)	1.0 (0.8–1.7)	7.6 (3.0–16.3)	1.6 (0.9–3.4)	1.9 (0.9–4.7)	1.0 (0.9–2.1)
Hospital length of stay (days), *median (IQR)*	11.6 (6.0–19.6)	8.4 (6.4–11.6)	16.5 (9.0–28.5)	11.1 (5.7–21.8)	11.2 (6.0–19.6)	8.9 (6.1–13.7)

The distribution of the EQ-5D-5L index scores before hospital admission and 1 year after ICU admission, and the distribution of the delta EQ-5D-5L index scores, are shown in Fig. E1.

Table 2 shows the changes in QoL 1 year after ICU for the different admission diagnoses. Of the 22 subgroups, 11 on average reported a clinically relevant improvement in QoL 1 year after ICU. In contrast, patients admitted to the ICU due to a cerebrovascular accident, intracerebral hemorrhage, trauma or neurotrauma, on average reported a decrease in QoL score. The largest average decrease in EQ-5D-5L index score was seen in trauma and neurotrauma patients (mean -0.13, SD 0.28), whereas the largest average increase was observed in patients admitted to the ICU due to COVID-19 (mean 0.18, SD 0.39).

**Table 2:** Changes in quality of life 1 year after ICU for different subgroups of ICU patients

Admission diagnosis	EQ-5D-5L index score before hospital admission *Median (IQR)*	EQ-5D-5L index score 1 year after ICU admission *Median (IQR)*	*P* value*	Delta EQ-5D-5L index score** Mean (SD)
*Cardiovascular (n* = *587, 15.0%)*	0.82 (0.59–0.96)	0.82 (0.70–0.91)	< 0.01	0.07 (0.30)
- Sepsis/septic shock (n = 230)	0.72 (0.45–0.88)	0.78 (0.64–0.88)	< 0.001	**0.11** (0.34)
- Hemodynamic instability (n = 168)	0.85 (0.70–1.00)	0.85 (0.76–1.00)	< 0.01	**0.09** (0.31)
- Cardiac arrest (n = 117)	1.00 (0.84–1.00)	0.87 (0.74–1.00)	< 0.001	-0.05 (0.19)
- Cardiac (n = 72)	0.78 (0.60–0.85)	0.81 (0.69–0.89)	0.02	**0.08** (0.24)
*Cardiovascular surgery (n* = *1464, 37.4%)*	0.83 (0.70–0.91)	0.86 (0.78–1.00)	< 0.001	0.07 (0.22)
- Thoracic aortic aneurysm (n = 172)	0.85 (0.74–0.91)	0.81 (0.74–0.89)	0.03	-0.02 (0.21)
- Coronary artery bypass grafting (n = 650)	0.85 (0.72–0.92)	0.89 (0.79–1.00)	< 0.001	**0.08** (0.22)
- Cardiac valve surgery (n = 506)	0.81 (0.66–0.89)	0.88 (0.78–1.00)	< 0.001	**0.09** (0.23)
- Vascular surgery (n = 136)	0.81 (0.66–0.89)	0.84 (0.74–0.90)	< 0.01	0.07 (0.22)
*Respiratory (n* = *675, 17.2%)*	0.68 (0.31–0.88)	0.81 (0.67–0.89)	< 0.001	**0.17** (0.38)
- Obstructive pulmonary disease (n = 39)	0.46 (0.17–0.70)	0.64 (0.37–0.76)	< 0.01	**0.16** (0.34)
- Pneumonia (n = 140)	0.63 (0.30–0.81)	0.73 (0.60–0.85)	< 0.001	**0.15** (0.32)
- COVID-19 (n = 440)	0.74 (0.33–0.89)	0.84 (0.72–0.91)	< 0.001	**0.18** (0.39)
- Other respiratory (n = 56)	0.74 (0.30–0.88)	0.79 (0.63–1.00)	0.01	**0.13** (0.43)
*Neurological (n* = *186, 4.7%)*	0.82 (0.55–0.91)	0.80 (0.62–0.89)	0.55	0.01 (0.36)
- Intracerebral hemorrhage (n = 67)	0.89 (0.76–1.00)	0.81 (0.63–0.88)	0.05	-**0.08** (0.29)
- Cerebrovascular accident (n = 20)	0.75 (0.62–0.86)	0.75 (0.54–0.83)	0.43	-**0.09** (0.42)
- Neurosurgery (n = 45)	0.78 (0.42–0.88)	0.73 (0.36–0.85)	0.53	0.02 (0.38)
- Other neurological (n = 54)	0.71 (0.45–0.91)	0.84 (0.66–0.91)	< 0.01	**0.15** (0.36)
*Trauma (n* = *184, 4.7%)*	1.00 (0.82–1.00)	0.82 (0.61–0.89)	< 0.001	-**0.13** (0.28)
- Neurotrauma (n = 87)	1.00 (0.85–1.00)	0.84 (0.65–0.92)	< 0.001	**-0.13** (0.26)
- Other trauma (n = 97)	1.00 (0.79–1.00)	0.81 (0.57–0.89)	< 0.001	-**0.13** (0.29)
*Other (n* = *817, 20.9%)*	0.81 (0.60–0.89)	0.82 (0.74–0.91)	< 0.001	**0.08** (0.26)
- Metabolic or endocrine (n = 41)	0.81 (0.63–0.89)	0.80 (0.67–0.89)	0.52	0.07 (0.32)
- Chest surgery (n = 110)	0.82 (0.66–0.91)	0.85 (0.78–0.98)	< 0.01	0.06 (0.21)
- Oncologic surgery (n = 385)	0.81 (0.66–0.89)	0.84 (0.74–0.92)	< 0.001	0.05 (0.23)
- Other (n = 281)	0.74 (0.51–0.86)	0.81 (0.70–0.89)	< 0.001	**0.12** (0.29)

*Comparison of EQ-5D-5L index score before hospital admission and EQ-5D-5L index score 1 year after ICU admission, using Wilcoxon signed rank test

**Clinically relevant changes in quality of life (≥ 0.08, based on MCID) are shown in bold

**Fig. 2:**
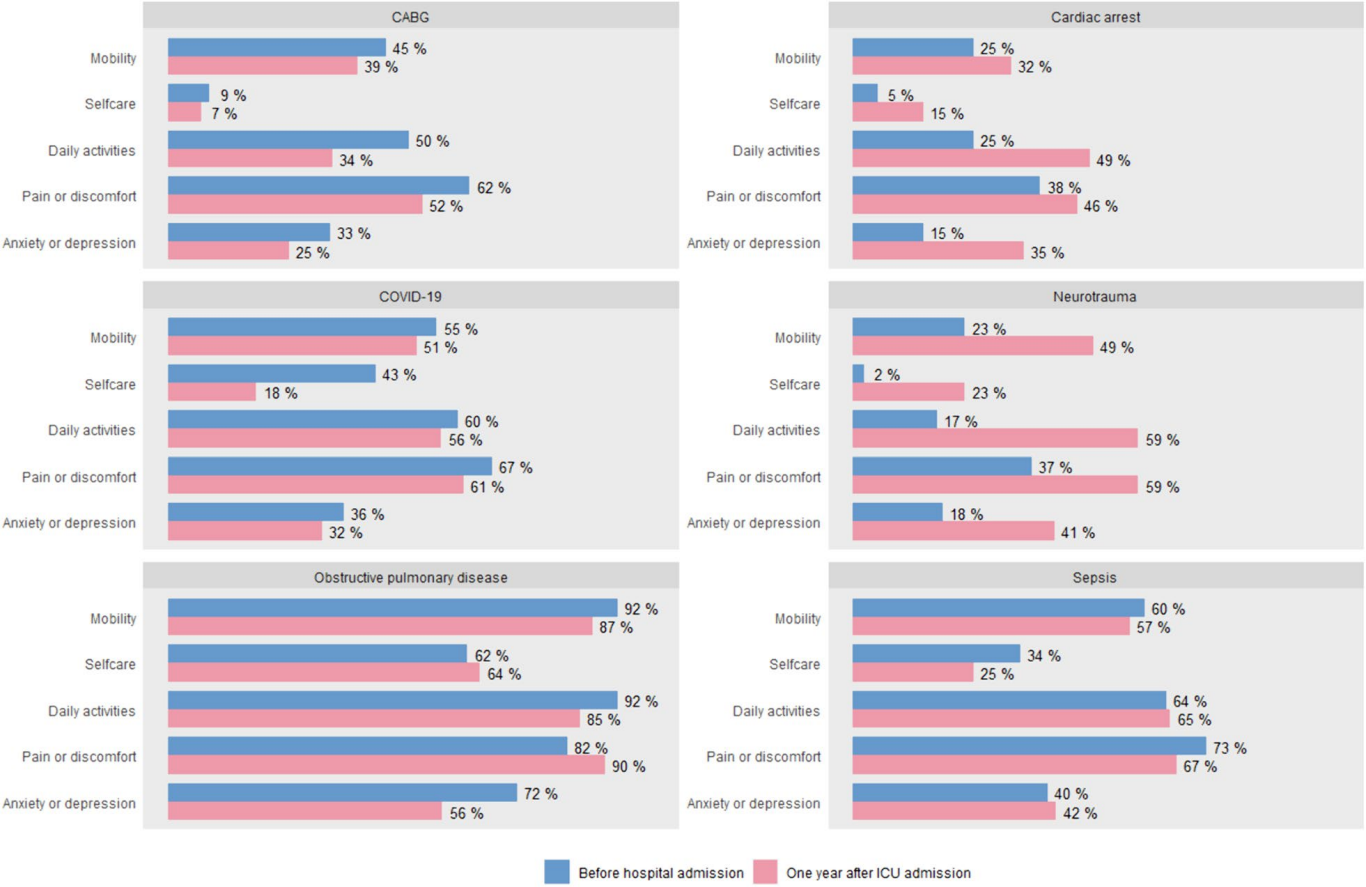
Percentage of patients reporting limitations in each dimension of the EQ-5D-5L, before hospital admission and 1 year after ICU admission

The lowest QoL 1 year after ICU admission was observed in the group admitted to the ICU due to obstructive pulmonary disease (EQ-5D-5L index score 0.64, IQR 0.37–0.76). This group also reported the lowest pre-admission QoL (EQ-5D-5L index score 0.46, IQR 0.17–0.70). Notably, before hospital admission 92.3% (n = 36) of these patients experienced limitations in their daily activities, while 82.1% (n = 32) experienced pain or discomfort. 1 year after ICU, this was true for 84.6% (n = 33) and 89.7% (n = 35), respectively. In contrast, before hospital admission, 2.3% (n = 2) of patients admitted due to neurotrauma experienced limitations in selfcare, while 17.2% (n = 15) experienced limitations in daily activities. 1 year after ICU admission, this increased to 23.0% (n = 20) and 58.6% (n = 51) respectively. The percentage of patients experiencing limitations in each dimension of the EQ-5D-5L, before and after ICU, is shown in Fig. 2 (for 6 subgroups) and Figure E2 (for the remaining subgroups).

**Fig. 3:**
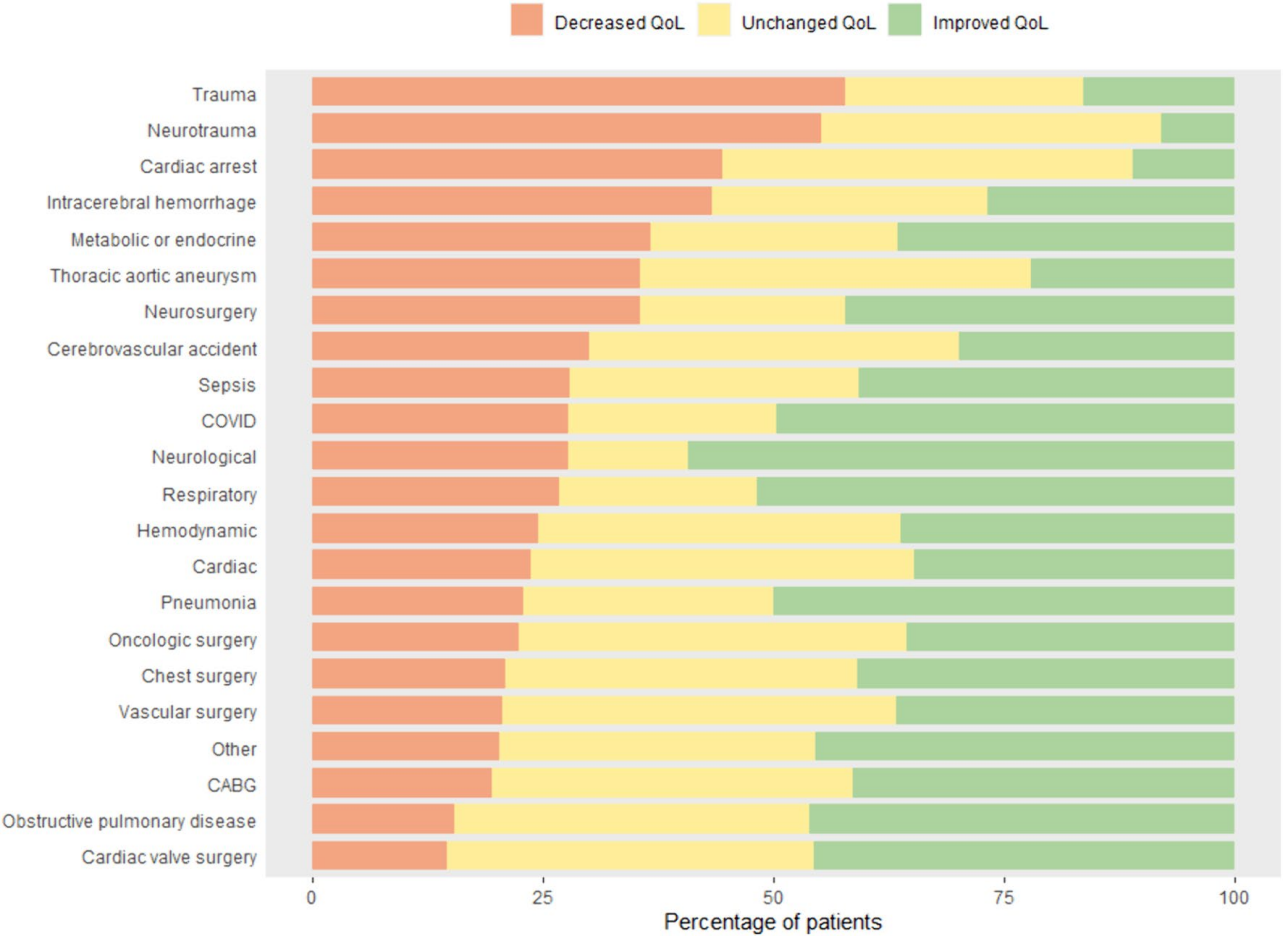
Percentage of patients experiencing an improved, unchanged or decreased quality of life 1 year after ICU admission, based on the MCID of 0.08

The highest QoL 1 year after ICU admission was seen in patients admitted after coronary artery bypass grafting (CABG) (EQ-5D-5L index score 0.89, IQR 0.79–1.00). In this group, 41.4% (n = 269) of patients reported an improvement in QoL 1 year after ICU, while after cardiac arrest this was true for 11.1% (n = 13) (Fig. 3). Moreover, after ICU admission due to a neurological trauma, more than half of patients (55.2%, n = 48) experienced a deterioration in QoL. However, in each of the studied 22 subgroups there were patients who reported a significant increase in QoL and patients who reported a significant decrease in QoL.

In general, 19.7% (n = 388) of patients admitted to the ICU after a planned surgery experienced a decrease in QoL, while 40.9% (n = 807) experienced an improvement in QoL. In contrast, after an acute ICU admission 31.0% (n = 601) reported a decrease in QoL, with a median EQ-5D-5L index score of 0.81 (IQR 0.67–0.89) 1 year post-ICU. For planned ICU admissions the median EQ-5D-5L index score 1 year post-ICU was 0.85 (IQR 0.75–1.00).

## Discussion

This large prospective multicenter cohort study including 3913 ICU survivors, showed that there is substantial variability in long-term QoL following ICU admission, even within subgroups. 1 year after ICU, patients admitted after a cerebrovascular accident, intracerebral hemorrhage and trauma, including neurotrauma, on average experienced a significant decrease in QoL. Conversely, 11 other subgroups, such as patients admitted after cardiac valve surgery, reported improvements in QoL 1 year after ICU.

Subgroups with the largest average improvements in QoL were patients admitted due to COVID-19, obstructive pulmonary disease, pneumonia and neurological disease. This improvement may be explained by the subacute nature of these diseases, as patients are asked to recall their QoL on the day before hospital admission, at which time they might already have been experiencing symptoms.

Another possible explanation is the phenomenon called “response shift”, which has previously been described in, among others, patients suffering from stroke and spinal cord injury [23–25]. This response shift is caused by psychological adaptation, altering the relationship between functional disabilities and subjective wellbeing. This could explain the discrepancy between the high incidence of physical, mental and cognitive symptoms after ICU, collectively called post-intensive care syndrome (PICS), and the relatively small number of patients reporting a decrease in QoL 1 year after ICU admission [6]. A previous study, showing that only the mental component of PICS is associated with a self-reported unacceptable outcome of ICU treatment, affirms this discrepancy between functional outcomes and subjective wellbeing [26]. This exhibits the importance of incorporating subjective outcomes, such as QoL, in decision-making.

However, up to now, only a few studies have assessed long-term outcomes in subgroups of ICU patients [1, 22, 27]. Importantly, very few of these studies have taken pre-admission QoL into account, while pre-admission QoL has been identified as the most important predictor of long-term outcomes after ICU [13, 14, 28]. Moreover, pre-admission QoL helps us put the impaired QoL of ICU survivors into perspective, as shown by this study. Compared to the general Dutch population, many patients reported lower EQ-5D-5L index scores, both before hospital admission and 1 year after ICU admission [15]. This finding suggests that this reduced QoL is not attributable to critical illness, as their pre-ICU QoL was already impaired.

To the best of our knowledge, this is the first study that explored changes in QoL in multiple subgroups of ICU survivors. This can give ICU clinicians insight into the long-term outcomes of critical illness in specific patient groups and help them to better inform patients and their family members about the long-term outcomes of critical illness.

However, this study does have some limitations. First, in case of an unplanned ICU admission, patients were asked to recall their QoL on the day before hospital admission, possibly leading to recall bias. Moreover, patients may already have been ill at the time, in which case the pre-admission QoL is potentially not representative of the pre-morbid QoL. Future studies should consider different reference points when measuring pre-admission QoL. Additionally, up to 35% of baseline questionnaires, depending on the admission diagnosis, were completed by proxies. However, studies have demonstrated that proxies are able to reliably assess patients’ quality of life [29–31]. Second, there were significant differences between responders and non-responders, with non-responders reporting a lower pre-ICU QoL and having more comorbidities. Furthermore, certain subgroups of ICU patients were more often lost to follow-up, due to either death (e.g., patients admitted to the ICU after oncologic surgery) or non-response (e.g., patients admitted to the ICU after trauma). This response bias may have resulted in an overestimation of the QoL of survivors. The same may be true for patients who did not wish to participate or were not included for other reasons, possibly resulting in selection bias. However, a recent study shows that the MONITOR-IC study participants have similar characteristics to the general Dutch ICU population [32]. Third, the EQ-5D-5L measures a patient’s limitations in five areas, possibly not capturing all components of QoL. Nonetheless, the EQ-5D-5L is a validated instrument, frequently used for measuring health-related QoL, both in the general population and after critical illness [12, 23]. Moreover, the EQ-5D-5L is practical due to its concise nature, and its importance is illustrated by its presence in core outcome sets [33–35]. Fourth, subgroups were based on admission diagnosis, which resulted in intuitive and clinically relevant subgroups. However, future studies could consider using artificial intelligence to create subgroups, as this might result in more homogenous patient groups.

## Conclusions

This large prospective multicenter cohort study demonstrated the diversity in long-term QoL between, and even within, subgroups of ICU survivors. These findings emphasize the need for personalized information and post-ICU care.

### Supplementary Information

Additional file1 (DOCX 8703 kb)

## Data Availability

The data that support the findings of this study are available from the corresponding author on reasonable request. The data are not publicly available due to them containing information that could compromise research participant privacy.

## Abbreviations

APACHE: Acute physiology and chronic health evaluation
CABG: Coronary artery bypass grafting
EQ-5D-5L: EuroQol five-dimensional
ICU: Intensive care unit
IQR: Interquartile range
MCID: Minimal clinically important difference
QoL: Quality of life
