# Supplementary material for: Changes in quality of life 1 year after intensive care: a multicenter prospective cohort of ICU survivors
Source: Crit Care. 2024 Jul 25;28:255. doi: 10.1186/s13054-024-05036-5 (PMC11271204; doi:10.1186/s13054-024-05036-5)

# ***Changes in Quality of Life One Year after Intensive Care; a Multicenter Prospective Cohort Study***

Supplemental material

1. E-Appendix 1: STROBE checklist
2. Table E1: list of diagnoses and subgroups
3. Table E2: responders vs non-responders
4. Figure E1: distribution of EQ-5D-5L index scores
5. Figure E2: percentage experiencing limitations in each dimension of EQ-5D-5L

**E-Appendix 1: STROBE checklist**

|  | Item No | Recommendation | Page No |
| --- | --- | --- | --- |
| **Title and abstract** | 1 | (*a*) Indicate the study’s design with a commonly used term in the title or the abstract | 1, 3 |
|  |  | (*b*) Provide in the abstract an informative and balanced summary of what was done and what was found |  |
| Introduction | | | |
| Background/rationale | 2 | Explain the scientific background and rationale for the investigation being reported | 5 |
| Objectives | 3 | State specific objectives, including any prespecified hypotheses | 5 |
| Methods | | | |
| Study design | 4 | Present key elements of study design early in the paper | 6 |
| Setting | 5 | Describe the setting, locations, and relevant dates, including periods of recruitment, exposure, follow-up, and data collection | 6-7 |
| Participants | 6 | (*a*) Give the eligibility criteria, and the sources and methods of selection of participants. Describe methods of follow-up | 6-7 |
|  |  | (*b*) For matched studies, give matching criteria and number of exposed and unexposed |  |
| Variables | 7 | Clearly define all outcomes, exposures, predictors, potential confounders, and effect modifiers. Give diagnostic criteria, if applicable | 7 |
| Data sources/ measurement | 8* | For each variable of interest, give sources of data and details of methods of assessment (measurement). Describe comparability of assessment methods if there is more than one group | 6-7 |
| Bias | 9 | Describe any efforts to address potential sources of bias | 6-7 |
| Study size | 10 | Explain how the study size was arrived at | 6, 9 |
| Quantitative variables | 11 | Explain how quantitative variables were handled in the analyses. If applicable, describe which groupings were chosen and why | 7-8 |
| Statistical methods | 12 | (*a*) Describe all statistical methods, including those used to control for confounding | 8 |
|  |  | (*b*) Describe any methods used to examine subgroups and interactions |  |
|  |  | (*c*) Explain how missing data were addressed |  |
|  |  | (*d*) If applicable, explain how loss to follow-up was addressed |  |
|  |  | (*e*) Describe any sensitivity analyses |  |
| Results | | |  |
| Participants | 13* | (a) Report numbers of individuals at each stage of study—eg numbers potentially eligible, examined for eligibility, confirmed eligible, included in the study, completing follow-up, and analysed | 9 |
|  |  | (b) Give reasons for non-participation at each stage |  |
|  |  | (c) Consider use of a flow diagram |  |
| Descriptive data | 14* | (a) Give characteristics of study participants (eg demographic, clinical, social) and information on exposures and potential confounders | 9 |
|  |  | (b) Indicate number of participants with missing data for each variable of interest |  |
|  |  | (c) Summarise follow-up time (eg, average and total amount) |  |
| Outcome data | 15* | Report numbers of outcome events or summary measures over time | 9-10 |

| Main results | 16 | (*a*) Give unadjusted estimates and, if applicable, confounder-adjusted estimates and their precision (eg, 95% confidence interval). Make clear which confounders were adjusted for and why they were included | 9-10 |
| --- | --- | --- | --- |
|  |  | (*b*) Report category boundaries when continuous variables were categorized |  |
|  |  | (*c*) If relevant, consider translating estimates of relative risk into absolute risk for a meaningful time period |  |
| Other analyses | 17 | Report other analyses done—eg analyses of subgroups and interactions, and sensitivity analyses | 10 |
| Discussion | | | |
| Key results | 18 | Summarise key results with reference to study objectives | 11 |
| Limitations | 19 | Discuss limitations of the study, taking into account sources of potential bias or imprecision. Discuss both direction and magnitude of any potential bias | 12 |
| Interpretation | 20 | Give a cautious overall interpretation of results considering objectives, limitations, multiplicity of analyses, results from similar studies, and other relevant evidence | 13 |
| Generalisability | 21 | Discuss the generalisability (external validity) of the study results | 12 |
| Other information | | | |
| Funding | 22 | Give the source of funding and the role of the funders for the present study and, if applicable, for the original study on which the present article is based | 2 |

*Give information separately for exposed and unexposed groups.

**Table E1**

Listed below are all APACHE IV diagnoses. Each diagnoses has been assigned to one of the 22 subgroups, listed in the right column.

| Diagnosis | Subgroup |
| --- | --- |
| Anaphylaxis | Hemodynamic instability |
| Aneurysm, dissecting aortic | Hemodynamic instability |
| Aneurysm/pesudoaneurysm, other | Hemodynamic instability |
| Angina, stable (asymp or stable pattern of symptoms w/meds) | Cardiac |
| Angina, unstable (angina interferes w/quality of life or meds are tolerated poorly) | Cardiac |
| Cardiac arrest (with or without respiratory arrest) | Cardiac arrest |
| Cardiomyopathy | Cardiac |
| Cardiovascular medical, other | Cardiac |
| Chest pain, atypical (noncardiac chest pain) | Other |
| Chest pain, epigastric | Other |
| Chest pain, musculoskeletal | Other |
| Chest pain, respiratory | Other |
| Chest pain, unknown origin | Other |
| CHF, congestive heart failure | Cardiac |
| Complications of previous open heart surgery (i.e. bleeding, infection etc.) | Other |
| Contusion, myocardial (include R/O) | Cardiac |
| Efffusion, pericardial | Cardiac |
| Endocarditis | Cardiac |
| Hematomas | Other |
| Hemorrhage (for gastrointestinal bleeding GI-see GI system) (for trauma see Trauma) | Hemodynamic instability |
| Hypertension, uncontrolled (for cerebrovascular accident-see Neurological System) | Hemodynamic instability |
| Hypovolemia (including dehydration. Do NOT include shock | Hemodynamic instability |
| Infarction, acute myocardial (MI), ANTERIOR | Cardiac |
| Infarction, acute myocardial (MI), INFEROLATERAL | Cardiac |
| Infarction, acute myocardial (MI), NON Q Wave | Cardiac |
| Infarction, acute myocardial (MI), none of the above | Cardiac |
| MI admitted > 24hrs after onset of ischemia | Cardiac |
| Monitoring, hemodynamic (pre-operative evaluation) | Hemodynamic instability |
| Papillary muscle rupture | Cardiac |
| Pericarditis | Cardiac |
| Rhythm disturbance (atrial, supraventricular) | Cardiac |
| Rhythm disturbance (conduction defect) | Cardiac |
| Rhythm disturbance (ventricular) | Cardiac |
| Sepsis, cutaneous/soft tissue | Sepsis/septic shock |
| Sepsis, GI | Sepsis/septic shock |
| Sepsis, gynecologic | Sepsis/septic shock |
| Sepsis, other | Sepsis/septic shock |
| Sepsis, pulmonary | Sepsis/septic shock |
| Sepsis, renal/UTI (including bladder) | Sepsis/septic shock |
| Sepsis, unknown | Sepsis/septic shock |
| Shock, cardiogenic | Hemodynamic instability |
| Tamponade, pericardial | Cardiac |
| Thrombosis, vascular (deep vein) | Other |
| Thrombus,arterial | Other |
| Toxicity, drug (i.e., beta blockers, calcium channel blockers, etc) | Hemodynamic instability |
| Vascular medical, other | Hemodynamic instability |
| Alcohol withdrawal | Other |
| Bleeding, GI from esophageal varices/portal hypertension | Hemodynamic instability |
| Bleeding, GI-location unknown | Hemodynamic instability |
| Bleeding, lower GI | Hemodynamic instability |
| Bleeding, upper GI | Hemodynamic instability |
| Cancer, colon/rectal | Other |
| Cancer, esophageal | Other |
| Cancer, other GI | Other |
| Cancer, pancreatic | Other |
| Cancer, stomach | Other |
| Cholangitis | Sepsis/septic shock |
| Diverticular disease | Sepsis/septic shock |
| Encephalopathy, hepatic | Neurological |
| GI Abscess/cyst | Sepsis/septic shock |
| GI medical, other | Other |
| GI Obstruction | Other |
| GI Perforation/rupture | Sepsis/septic shock |
| GI Vascular insufficiency | Other |
| Hepatic failure, acute | Metabolic/Endocrine |
| Inflammatory bowel disease | Other |
| Pancreatitis | Sepsis/septic shock |
| Peritonitis | Sepsis/septic shock |
| Ulcer disease, peptic | Other |
| Genitourinary medical, other | Other |
| Hemorrhage, postpartum | Hemodynamic instability |
| Hepato-renal syndrome | Metabolic/Endocrine |
| Pre-eclampsia/eclampsia | Metabolic/Endocrine |
| Renal bleeding | Hemodynamic instability |
| Renal failure, acute | Metabolic/Endocrine |
| Renal infection/abscess | Sepsis/septic shock |
| Renal neoplasm, cancer | Other |
| Renal obstruction | Other |
| Anemia | Other |
| Blood transfusion reaction | Other |
| Coagulopathy | Other |
| Hematologic medical, other | Other |
| Leukemia, acute lymphocytic | Other |
| Leukemia, acute myelocytic | Other |
| Leukemia, chronic lymphocytic | Other |
| Leukemia, chronic myelocytic | Other |
| Leukemia, other | Other |
| Lymphoma, Hodgkins | Other |
| Lymphoma, non-Hodgkins | Other |
| Neutropenia | Other |
| Pancytopenia | Other |
| Sickle cell crisis | Other |
| Thrombocytopenia | Other |
| Acid-base electrolyte disturbance | Metabolic/Endocrine |
| Addisons disease | Metabolic/Endocrine |
| Adrenal neoplasm (including pheochromocytoma) | Other |
| Diabetic hyperglycemic hyperosmolar nonketotic coma (HHNC) | Metabolic/Endocrine |
| Diabetic ketoacidosis | Metabolic/Endocrine |
| Heat exhaustion/stroke | Metabolic/Endocrine |
| Hyperthermia | Metabolic/Endocrine |
| Hyperthyroid storm/crisis | Metabolic/Endocrine |
| Hypoglycemia | Metabolic/Endocrine |
| Hypothermia | Metabolic/Endocrine |
| Hypothyroid/myxedema | Metabolic/Endocrine |
| Metabolic/endocrine medical, other | Metabolic/Endocrine |
| Thyroid neoplasm | Other |
| Arthritis, rheumatoid | Other |
| Arthritis, septic | Sepsis/septic shock |
| Burn | Other |
| Cellulitis and localized soft tissue infections | Sepsis/septic shock |
| Connective tissue disease (mixed) | Other |
| Lupus, systemic | Other |
| Musculoskeletal medical, other | Other |
| Myositis, viral | Other |
| Rhabdomyolysis | Other |
| Scleroderma | Other |
| Vasculitis | Other |
| Abscess, neurologic | Neurological |
| Amyotrophic lateral sclerosis | Other |
| Coma/change in level of consciousness (for hepatic see GI, for diabetic see Endocrine) | Neurological |
| CVA, cerebrovascular accident/stroke | Cerebrovascular accident |
| Drug withdrawal | Other |
| Encephalitis | Neurological |
| Encephalopathies (excluding hepatic) | Neurological |
| Guillian-Barre syndrome | Neurological |
| Hematoma, epidural | Neurotrauma |
| Hematoma, subdural | Neurotrauma |
| Hemorrhage/hematoma, intracranial | Intracerebral hemorrhage |
| Hydrocephalus, obstructive | Neurological |
| Meningitis | Neurological |
| Myasthenia gravis | Neurological |
| Neoplasm, neurologic | Neurological |
| Neurologic medical, other | Neurological |
| Neuromuscular medical, other | Neurological |
| Nontraumatic coma due to anoxia/ischemia | Neurological |
| Overdose, alcohols (ethanol, methanol, ethylene glycol) | Other |
| Overdose, analgesic (aspirin, cetaminophen) | Other |
| Overdose, antidepressants (cyclic, lithium) | Other |
| Overdose, other toxin, poison or drug | Other |
| Overdose, sedatives, hypnotics, antipsychotics, benzodiazepines | Other |
| Overdose, street drugs (opiates, cocaine, amphetamine) | Other |
| Poisoning, carbon monoxide, arsenic, cyanide | Other |
| Seizures (primary-no structural brain disease) | Neurological |
| Subarachnoid hemorrhage/arteriovenous malformation | Intracerebral hemorrhage |
| Subarachnoid hemorrhage/intracranial aneurysm | Intracerebral hemorrhage |
| Apnea, sleep | Respiratory |
| ARDS-adult respiratory distress syndrome, non-cardiogenic pulmonary edema | Respiratory |
| Arrest, respiratory (without cardiac arrest) | Respiratory |
| Asthma | Obstructive pulmonary disease |
| Atelectasis | Respiratory |
| Cancer, laryngeal | Other |
| Cancer, lung | Other |
| Cancer, oral | Other |
| Cancer, tracheal | Other |
| Effusions, pleural | Respiratory |
| Embolus, pulmonary | Hemodynamic instability |
| Emphysema/bronchitis | Obstructive pulmonary disease |
| Hemorrhage/hemoptysis, pulmonary | Respiratory |
| Hemothorax | Respiratory |
| Hypertension-pulmonary, primary/idiopathic | Respiratory |
| Near drowning accident | Respiratory |
| Obstruction-airway (i.e. acute epiglottitis, post-extubation edema, foreign body, etc.) | Other |
| Pneumonia, aspiration | Pneumonia |
| Pneumonia, bacterial | Pneumonia |
| Pneumonia, fungal | Pneumonia |
| Pneumonia, other | Pneumonia |
| Pneumonia, parasitic (i.e. Pneumocystis pneumonia) | Pneumonia |
| Pneumonia, viral | Pneumonia |
| Pneumothorax | Respiratory |
| Respiratory- medical, other | Respiratory |
| Restrictive lung disease (i.e. sarcoidosis, pulmonary fibrosis) | Respiratory |
| Smoke inhalation | Respiratory |
| Weaning from mechanical ventilation (transfer from other unit or hospital only) | Other |
| Bone marrow transplant, non operative admission | Other |
| Heart transplant, non operative admission | Other |
| Heart-lung transplant, non operative admission | Other |
| Kidney transplant, non operative admission | Other |
| Kidney-pancreas transplant, non operative admission | Other |
| Liver transplant, non operative admission | Other |
| Liver-small bowel transplant, non operative admission | Other |
| Lung transplant, bilateral, non operative admission | Other |
| Lung transplant, single, non operative admission | Other |
| Pancreas transplant, non operative admission | Other |
| Small bowel transplant, non operative admission | Other |
| Transplant, other, non operative admission | Other |
| Abdomen only trauma | Other trauma |
| Abdomen/extremity trauma | Other trauma |
| Abdomen/face trauma | Other trauma |
| Abdomen/multiple trauma | Other trauma |
| Abdomen/pelvis trauma | Other trauma |
| Abdomen/spinal trauma | Other trauma |
| Chest/abdomen trauma | Other trauma |
| Chest/extremity trauma | Other trauma |
| Chest/face trauma | Other trauma |
| Chest/multiple trauma | Other trauma |
| Chest/pelvis trauma | Other trauma |
| Chest/spinal trauma | Other trauma |
| Chest/thorax only trauma | Other trauma |
| Estremity only trauma | Other trauma |
| Extremity/face trauma | Other trauma |
| Extremity/multiple trauma | Other trauma |
| Face only trauma | Other trauma |
| Face/multiple trauma | Other trauma |
| Head (CNS) only trauma | Neurotrauma |
| Head/abdomen trauma | Neurotrauma |
| Head/chest trauma | Neurotrauma |
| Head/extremity trauma | Neurotrauma |
| Head/face trauma | Neurotrauma |
| Head/multiple trauma | Neurotrauma |
| Head/pelvis trauma | Neurotrauma |
| Head/spinal trauma | Neurotrauma |
| Pelvis/extremity trauma | Other trauma |
| Pelvis/face trauma | Other trauma |
| Pelvis/hip only trauma | Other trauma |
| Pelvis/multiple trauma | Other trauma |
| Pelvis/spinal trauma | Other trauma |
| Spinal cord only trauma | Other trauma |
| Spinal/extremity trauma | Other trauma |
| Spinal/face trauma | Other trauma |
| Spinal/multiple trauma | Other trauma |
| Trauma medical, other | Other trauma |
| Ablation or mapping of cardiac conduction pathway | Chest surgery |
| Aneurysm repair, ventricular | Chest surgery |
| Aneurysm, abdominal aortic | Vascular surgery |
| Aneurysm, abdominal aortic; with dissection | Vascular surgery |
| Aneurysm, abdominal aortic; with rupture | Vascular surgery |
| Aneurysm, thoracic aortic | Thoracic aortic aneurysm |
| Aneurysm, thoracic aortic; with dissection | Thoracic aortic aneurysm |
| Aneurysm, thoracic aortic; with rupture | Thoracic aortic aneurysm |
| Aneurysms, repair of other (except ventricular) | Other |
| Aortic and Mitral valve replacement | Cardiac valve surgery |
| Aortic valve replacement (isolated) | Cardiac valve surgery |
| Atrial Septal Defect (ASD) Repair | Chest surgery |
| CABG alone, coronary artery bypass grafting | CABG |
| CABG alone, redo | CABG |
| CABG redo with other operation | CABG |
| CABG redo with valve repair/replacement | Cardiac valve surgery |
| CABG with aortic valve replacement | Cardiac valve surgery |
| CABG with double valve repair/replacement | Cardiac valve surgery |
| CABG with mitral valve repair | Cardiac valve surgery |
| CABG with mitral valve replacement | Cardiac valve surgery |
| CABG with other operation | CABG |
| CABG with pulmonic or tricuspid valve repair or replacement ONLY. | Cardiac valve surgery |
| CABG, Minimally invasive; Mid-CABG | CABG |
| Cardiovascular surgery, other | Chest surgery |
| Complications of prev. peripheral vasc. surgery,surgery for (i.e.ligation of bleeder, exploration and evacuation of hematoma, debridement, pseudoaneurysms, clots, fistula, etc.) | Other |
| Complications of previous open-heart surgery, surgery for (i.e. bleeding, infection, mediastinal rewiring,leaking aortic graft etc.) | Other |
| Congenital Defect Repair (Other) | Chest surgery |
| Defibrillator, automatic implantable cardiac; insertion of | Other |
| Dilatation (with general anesthesia) | Vascular surgery |
| Dilatation (without general anesthesia) | Vascular surgery |
| Embolectomy (with general anesthesia) | Vascular surgery |
| Embolectomy (without general anesthesia) | Vascular surgery |
| Endarterectomy (other vessels) | Vascular surgery |
| Endarterectomy, carotid | Vascular surgery |
| Graft for dialysis, insertion of | Vascular surgery |
| Graft, aorto-femoral bypass | Vascular surgery |
| Graft, aorto-iliac bypass | Vascular surgery |
| Graft, femoral-femoral bypass | Vascular surgery |
| Graft, femoral-popliteal bypass | Vascular surgery |
| Grafts, all other bypass (except renal) | Vascular surgery |
| Grafts, all renal bypass | Vascular surgery |
| Grafts, removal of infected vascular | Vascular surgery |
| Mitral valve repair | Cardiac valve surgery |
| Mitral valve replacement | Cardiac valve surgery |
| Pericardial effusion/tamponade | Chest surgery |
| Pericardiectomy (total/subtotal) | Chest surgery |
| Pulmonary valve surgery | Cardiac valve surgery |
| Thrombectomy (with general anesthesia) | Vascular surgery |
| Thrombectomy (without general anesthesia) | Vascular surgery |
| Tricuspid valve surgery | Cardiac valve surgery |
| Tumor removal, intracardiac | Chest surgery |
| Vascular surgery, other | Vascular surgery |
| Vena cava clipping | Chest surgery |
| Vena cava filter insertion | Chest surgery |
| Ventricular Septal Defect (VSD) Repair | Chest surgery |
| Appendectomy | Sepsis/septic shock |
| Bleeding-lower GI, surgery for | Hemodynamic instability |
| Bleeding-other GI, surgery for | Hemodynamic instability |
| Bleeding-upper GI, surgery for | Hemodynamic instability |
| Bleeding-variceal, surgery for (excluding vascular shuntingsee surgery for portosystemic shunt) | Hemodynamic instability |
| Cancer-colon/rectal, surgery for (including abdominoperineal resections) | Oncologic surgery |
| Cancer-esophageal, surgery for (abdominal approach) | Oncologic surgery |
| Cancer-other GI tract, surgery for (i.e. hepatoma, gallbladder etc.) | Oncologic surgery |
| Cancer-small intestinal, surgery for | Oncologic surgery |
| Cancer-stomach, surgery for | Oncologic surgery |
| CAPD catheter insertion | Other |
| Cholecystectomy/cholangitis, surgery for (gallbladder removal) | Sepsis/septic shock |
| Complications of previous GI surgery; surgery for (anastomotic leak, bleeding, abscess, infection, dehiscence, etc.) | Other |
| Diverticular disease, surgery for | Sepsis/septic shock |
| Esophageal surgery, other | Chest surgery |
| Fistula/abscess, surgery for (not inflammatory bowel disease) | Other |
| Gastrostomy | Other |
| GI Abscess/cyst-primary, surgery for (for complications of GI surgery see below) | Sepsis/septic shock |
| GI Obstruction, surgery for (including lysis of adhesions) | Sepsis/septic shock |
| GI Perforation/rupture, surgery for | Sepsis/septic shock |
| GI surgery, other | Other |
| GI Vascular ischemia, surgery for (resection) | Sepsis/septic shock |
| Hemorrhage, intra/retroperitoneal | Hemodynamic instability |
| Hernia-hiatal, esophageal surgery for | Chest surgery |
| Herniorrhaphy | Other |
| Inflammatory bowel disease, surgery for | Sepsis/septic shock |
| Obesity-morbid, surgery for | Other |
| Pancreatitis, surgery for | Sepsis/septic shock |
| Peritoneal lavage | Other |
| Peritonitis, surgery for | Sepsis/septic shock |
| Shunt, peritoneal-venous;surgery for | Other |
| Shunt-portosystemic, surgery for | Other |
| Splenectomy | Other |
| Whipple-surgery for pancreatic cancer | Oncologic surgery |
| Bladder repair for perforation/rupture | Other |
| Cesarean section | Other |
| Cyst, ruptured ovarian | Other |
| Cystectomy (other reasons) | Other |
| Cystectomy for neoplasm | Oncologic surgery |
| Ectopic pregnancy (all) | Other |
| Exenteration, pelvic -male | Other |
| Exenteration, pelvic-female | Oncologic surgery |
| Genitourinary surgery, other | Other |
| Hysterectomy for cancer with or without lymph node dissection | Oncologic surgery |
| Hysterectomy for other benign neoplasm/fibroids | Other |
| Lymph node dissection, pelvic or retroperitoneal(female) | Oncologic surgery |
| Lymph node dissection, pelvic or retroperitoneal(male) | Oncologic surgery |
| Mastectomy (all) | Oncologic surgery |
| Nephrectomy (other reasons) | Other |
| Nephrectomy for neoplasm | Oncologic surgery |
| Obstruction due to neoplasm ,surgery for; (with or without ilealconduit) | Oncologic surgery |
| Obstruction due to nephrolithiasis, surgery for (with or without ileal-conduit) | Other |
| Obstruction/other, surgery for (with or without ileal-conduit) | Other |
| Oophorectomy with/without salpingectomy with/without lymph node dissection | Oncologic surgery |
| Orchiectomy with/without pelvic lymph node dissection | Oncologic surgery |
| Pelvic relaxation (cystocele, rectocele, etc.) | Other |
| Prostatectomy, suprapubic; for benign prostatic hypertrophy | Other |
| Prostatectomy, suprapubic; for cancer | Oncologic surgery |
| TURP, transurethral prostate resection for benign prostatic hypertrophy | Other |
| TURP, transurethral prostate resection for cancer | Oncologic surgery |
| Hematologic surgery, other | Other |
| Lymphoma Hodgkins, surgery for (including staging) | Other |
| Lymphoma, non-Hodgkins; surgery for (including staging) | Other |
| Metabolic/endocrine surgery, other | Other |
| Parathyroidectomy | Other |
| Thyroidectomy | Other |
| Thyroidectomy and parathyoidectomy | Other |
| Adrenalectomy | Other |
| Amputation (non-traumatic) | Other |
| Cellulitis and localized soft tissue infections, surgery for | Sepsis/septic shock |
| Cosmetic surgery (all) | Other |
| Fracture-pathological, non-union, non-traumatic, for fractures due to trauma see Trauma | Other |
| Grafting, skin (all) | Other |
| Hip replacement, total (non-traumatic) | Other |
| Knee replacement, total (non-traumatic) | Other |
| Orthopedic surgery, other | Other |
| Skin surgery, other | Other |
| Abscess/infection-cranial, surgery for | Neurosurgery |
| Anastomosis, vascular | Other |
| Arteriovenous malformation, surgery for | Neurosurgery |
| Biopsy, brain | Neurosurgery |
| Burr hole placement | Other |
| Cerebrospinal fluid leak, surgery for | Other |
| Complications of previous spinal cord surgery, surgery for | Other |
| Cranial nerve, decompression/ligation | Neurosurgery |
| Cranioplasty and complications from previous craniotomies | Other |
| Devices for spine fracture/dislocation | Other |
| Fusion-spinal/Harrington rods | Neurosurgery |
| Hematoma, epidural, surgery for | Neurotrauma |
| Hematoma, subdural, surgery for | Neurotrauma |
| Hemorrhage/hematoma-intracranial, surgery for | Intracerebral hemorrhage |
| Laminectomy/spinal cord decompression (excluding malignancies) | Neurosurgery |
| Neoplasm-cranial, surgery for (excluding transphenoidal) | Oncologic surgery |
| Neoplasm-spinal cord surgery or other related procedures | Oncologic surgery |
| Neurologic surgery, other | Neurosurgery |
| Seizures-intractable, surgery for | Other |
| Shunts and revisions | Other |
| Spinal cord sugery, other | Other |
| Stereotactic procedure | Other |
| Subarachnoid hemorrhage/intracranial aneurysm, surgery for | Intracerebral hemorrhage |
| Sympathectomy | Other |
| Transphenoidal surgery | Neurosurgery |
| Ventriculostomy | Neurosurgery |
| Apnea-sleep; surgery for (i.e. UPPP-uvulopalatopharyngoplasty) | Other |
| Biopsy, open lung | Chest surgery |
| Bullectomy | Chest surgery |
| Cancer oral/sinus, surgery for | Oncologic surgery |
| Cancer-laryngeal/tracheal, surgery for | Oncologic surgery |
| Facial surgery (if related to trauma, see Trauma) | Other |
| Infection/abscess, other surgery for | Sepsis/septic shock |
| Respiratory surgery, other | Chest surgery |
| Thoracotomy for benign tumor (i.e. mediastinal chest wall mass, thymectomy) | Chest surgery |
| Thoracotomy for bronchopleural fistula | Chest surgery |
| Thoracotomy for esophageal cancer | Oncologic surgery |
| Thoracotomy for lung cancer | Oncologic surgery |
| Thoracotomy for lung reduction | Chest surgery |
| Thoracotomy for other malignancy in chest | Oncologic surgery |
| Thoracotomy for other reasons | Chest surgery |
| Thoracotomy for pleural disease | Chest surgery |
| Thoracotomy for thoracic/respiratory infection | Sepsis/septic shock |
| Tracheostomy | Other |
| Bone marrow transplant | Other |
| Heart transplant | Other |
| Heart-lung transplant | Other |
| Kidney transplant | Other |
| Kidney-pancreas transplant | Other |
| Liver transplant | Other |
| Liver-small bowel transplant | Other |
| Lung transplant, bilateral | Other |
| Lung transplant, single | Other |
| Pancreas transplant | Other |
| Small bowel transplant | Other |
| Transplant, other | Other |
| Abdomen only trauma, surgery for | Other trauma |
| Abdomen/extremity trauma, surgery for | Other trauma |
| Abdomen/face trauma, surgery for | Other trauma |
| Abdomen/multiple trauma, surgery for | Other trauma |
| Abdomen/pelvis trauma, surgery for | Other trauma |
| Abdomen/spinal trauma, surgery for | Other trauma |
| Chest/abdomen trauma, surgery for | Other trauma |
| Chest/extremity trauma, surgery for | Other trauma |
| Chest/face trauma, surgery for | Other trauma |
| Chest/multiple trauma, surgery for | Other trauma |
| Chest/pelvis trauma, surgery for | Other trauma |
| Chest/spinal trauma, surgery for | Other trauma |
| Chest/thorax only trauma, surgery for | Other trauma |
| Extremity only trauma, surgery for | Other trauma |
| Extremity/face trauma, surgery for | Other trauma |
| Extremity/multiple trauma, surgery for | Other trauma |
| Face only trauma, surgery for | Other trauma |
| Face/multiple trauma, surgery for | Other trauma |
| Head (CNS) only trauma, surgery for | Neurotrauma |
| Head/abdomen trauma, surgery for | Neurotrauma |
| Head/chest trauma, surgery for | Neurotrauma |
| Head/extremity trauma, surgery for | Neurotrauma |
| Head/face trauma, surgery for | Neurotrauma |
| Head/multiple trauma, surgery for | Neurotrauma |
| Head/pelvis trauma, surgery for | Neurotrauma |
| Head/spinal trauma, surgery for | Neurotrauma |
| Pelvis/extremity trauma, surgery for | Other trauma |
| Pelvis/face trauma, surgery for | Other trauma |
| Pelvis/hip only trauma,surgery for | Other trauma |
| Pelvis/multiple trauma, surgery for | Other trauma |
| Pelvis/spinal trauma, surgery for | Other trauma |
| Spinal cord only trauma, surgery for | Other trauma |
| Spinal/extremity trauma, surgery for | Other trauma |
| Spinal/face trauma, surgery for | Other trauma |
| Spinal/multiple trauma, surgery for | Other trauma |
| Trauma surgery, other | Other trauma |

**Table E2**

Baseline characteristics responders, defined as patients who returned 1-year follow-up questionnaire, versus non-responders, and baseline characteristics of patients who died in the follow-up period

|  | Responders  (n = 4150) | Non-responders  (n = 1048) | p-value* | Died  (n = 336) |
| --- | --- | --- | --- | --- |
| Sex: Female, *n (%)* | 1299 (31.3%) | 413 (39.4%) | <0.001 | 107 (31.9%) |
| Age (years), *mean (SD)* | 62.8 (12.6) | 59.9 (15.8) | <0.001 | 67.1 (11.1) |
| Pre-admission quality of life (EQ-5D-5L), *median (IQR)* | 0.81 (0.61 – 0.91) | 0.77 (0.51 – 0.89) | <0.001 | 0.71 (0.43 – 0.86) |
| Chronic Obstructive Pulmonary Disease, *n (%)* | 393 (9.5%) | 121 (11.6%) | 0.04 | 56 (16.7%) |
| Diabetes, *n (%)* | 523 (12.6%) | 158 (15.1%) | 0.03 | 55 (16.4%) |
| Chronic renal insufficiency, *n (%)* | 124 (3.0%) | 37 (3.5%) | 0.4 | 24 (7.1%) |
| Admission type, *n (%)*   - Medical - Acute surgical - Planned surgical | 1622 (39.1%)  463 (11.2%)  2064 (49.8%) | 438 (41.8%)  133 (12.7%)  476 (45.5%) | 0.04 | 142 (42.3%)  30 (8.9%)  164 (48.8%) |
| APACHE diagnosis group   - Cardiovascular - Cardiovascular surgery - Neurological - Respiratory - Trauma - Other | 622 (15.0%)  1540 (37.1%)  192 (4.6%)  725 (17.5%)  199 (4.8%)  872 (21.0%) | 176 (16.8%)  303 (28.9%)  79 (7.5%)  149 (14.2%)  73 (7.0%)  268 (25.6%) | <0.001 | 63 (18.8%)  41 (12.2%)  17 (5.1%)  45 (13.4%)  15 (4.5%)  155 (46.1%) |
| Mechanical ventilation in first 24 hours, *n (%)* | 2956 (71.2%) | 667 (63.7%) | <0.001 | 204 (60.7%) |
| Use of vasoactive medication in first 24 hours, *n (%)* | 2541 (61.2%) | 545 (52.0%) | <0.001 | 201 (59.8%) |
| APACHE IV score, *mean (SD)* | 55.7 (20.9) | 55.0 (23.8) | 0.03 | 63.8 (26.5) |
| Hospital length of stay (days), *median (IQR)* | 9.6 (6.6 – 16.0) | 9.5 (6.0 – 15.6) | 0.2 | 12.5 (7.6 – 22.5) |
| ICU length of stay (days), *median (IQR)* | 1.5 (0.9 – 3.7) | 1.3 (0.9 – 3.4) | 0.2 | 2.0 (1.0 – 4.8) |

*Comparison of responders and non-responders, using chi-square test, independent-sample t-test, or Wilcoxon rank sum test, whenever appropriate

**Figure E1**

Distribution of quality of life before hospital admission, quality of life 1 year after ICU admission, and change in quality of life 1 year after ICU admission (n = 3913)

**
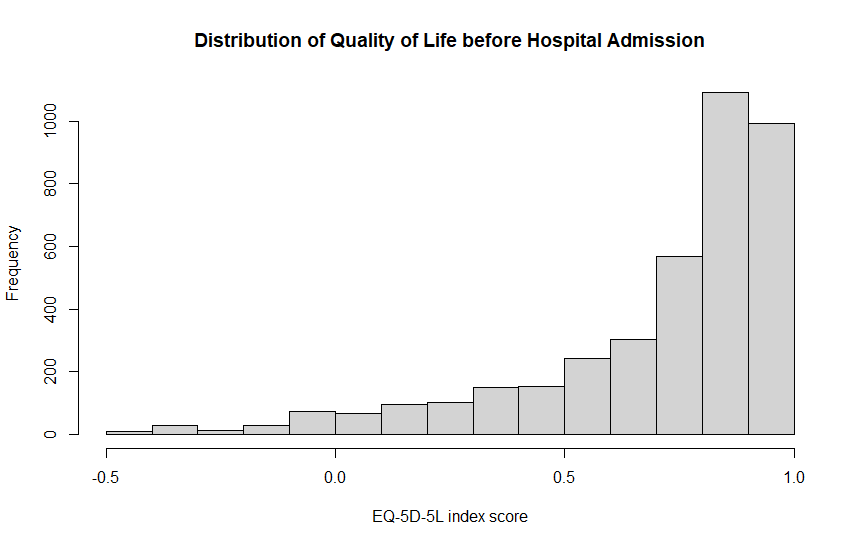
**

**
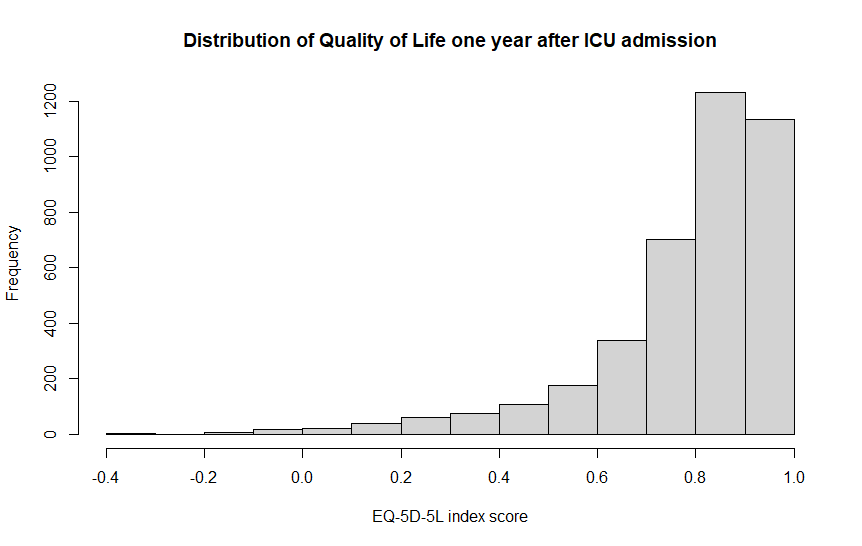
**

**
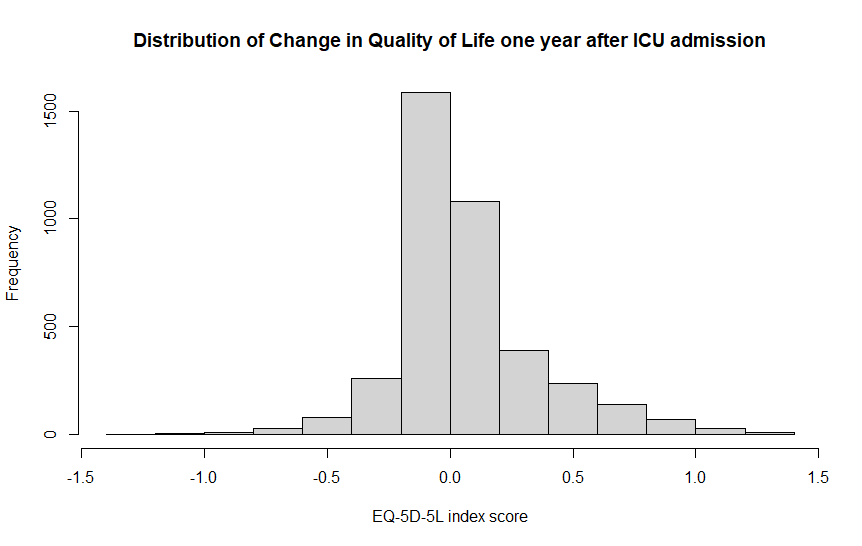
**

**Figure E2**

Percentage of patients experiencing limitations in each dimension of the EQ-5D-5L, before and after ICU.

**Cardiovascular subgroups**


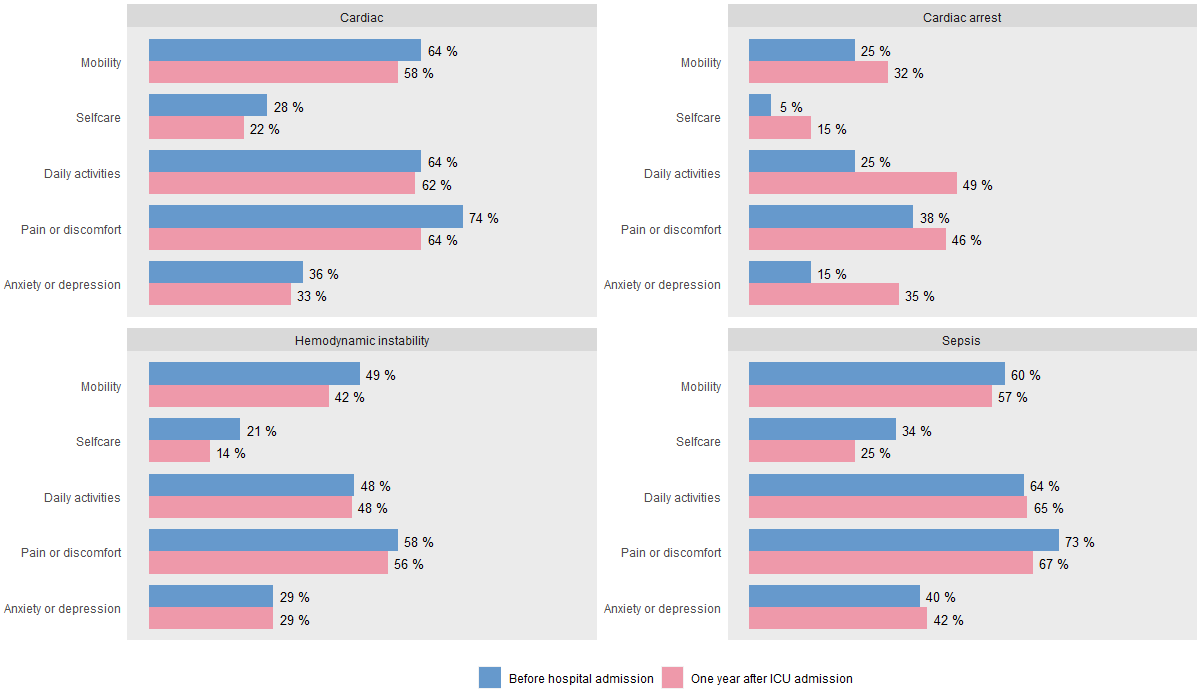


**Cardiovascular surgery subgroups**

**
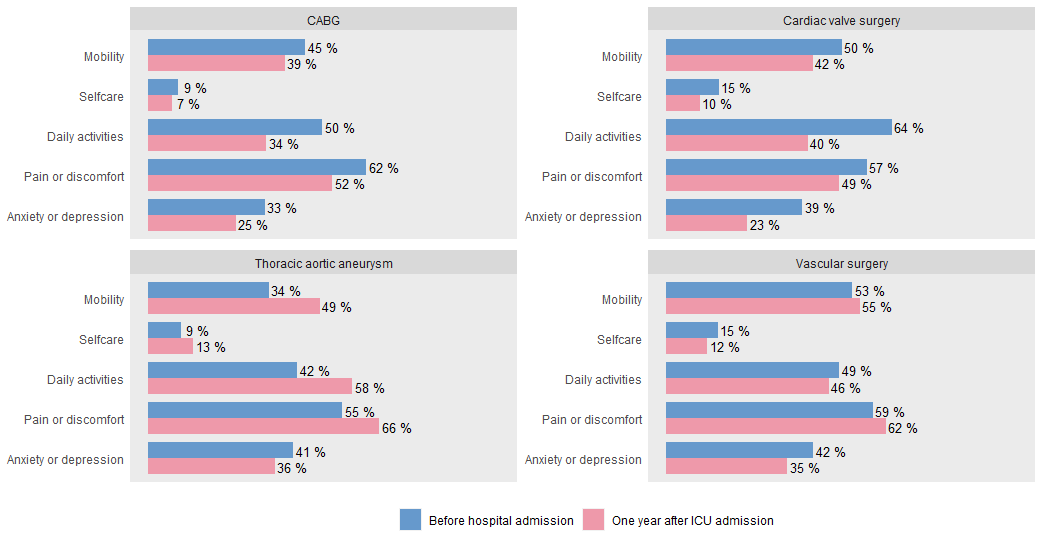
**

**Respiratory subgroups**

**
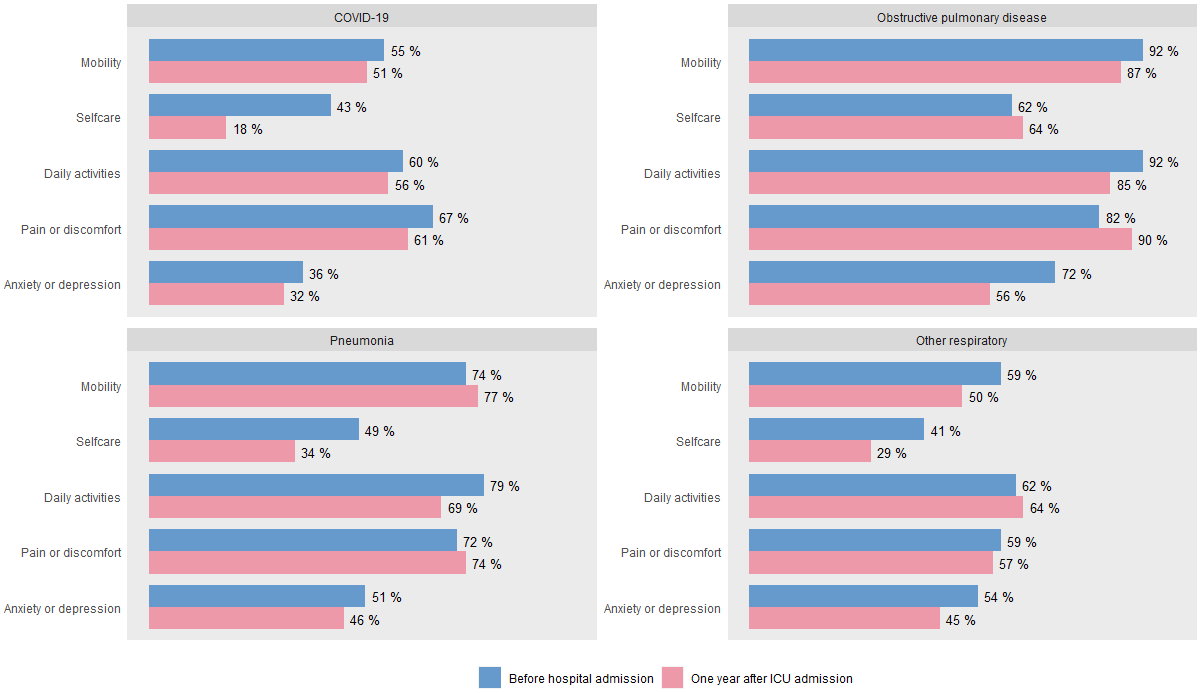
**

**Neurological subgroups**

**
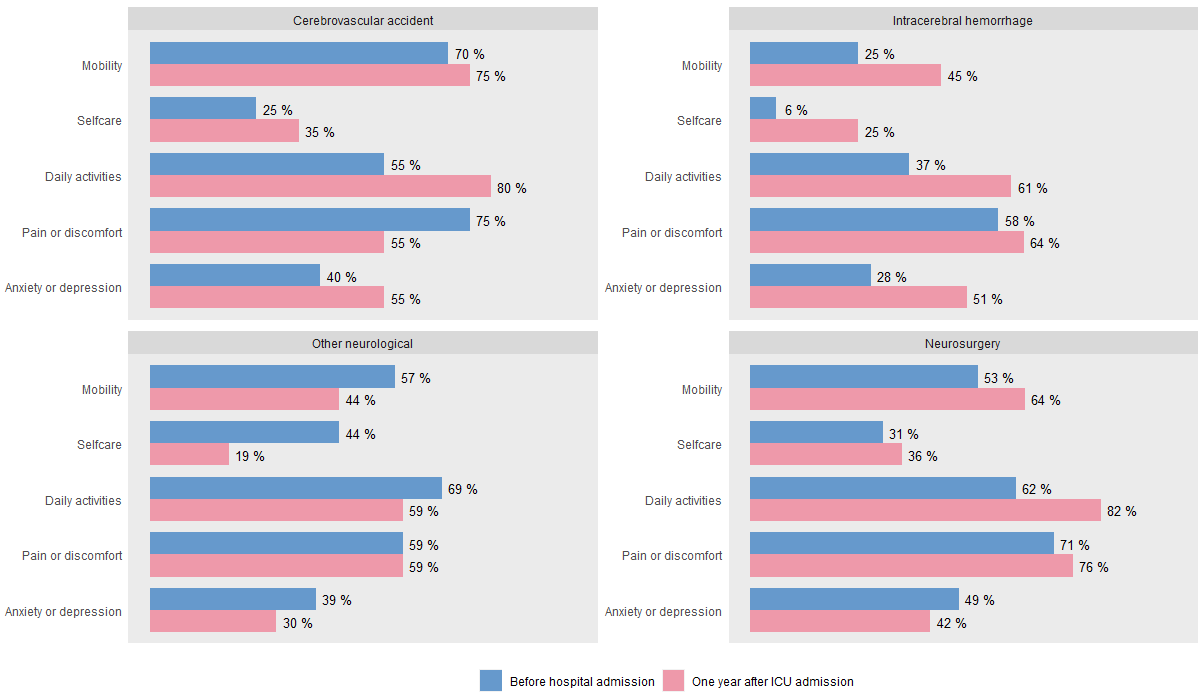
**

**Trauma subgroups**


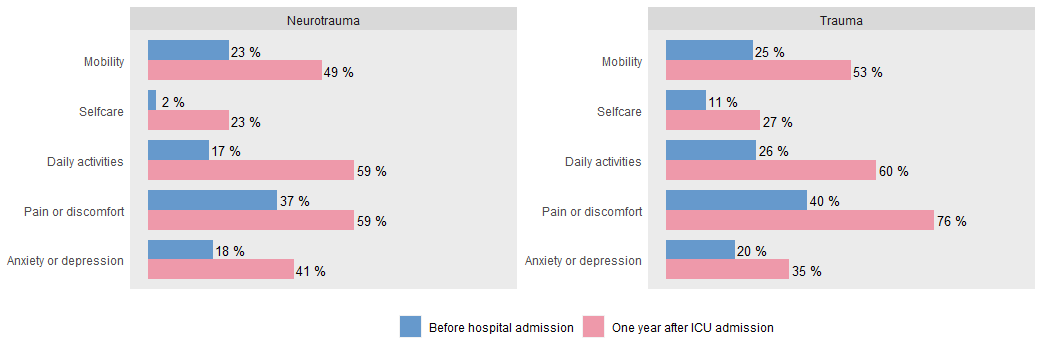


**Other subgroups**


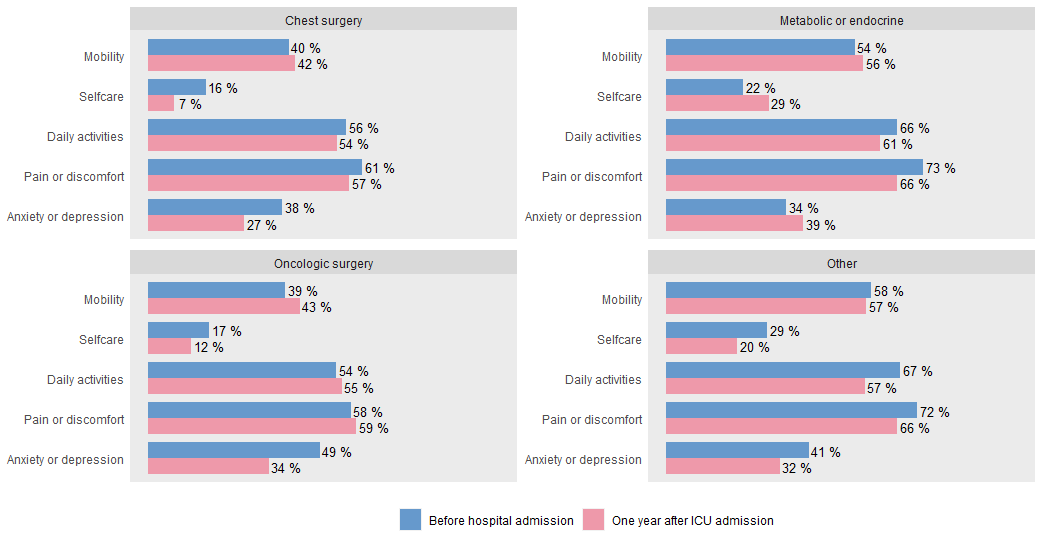

Supplement: Supplementary file 1 — Additional file1 (DOCX 8703 kb) [file 13054_2024_5036_MOESM1_ESM.docx]
